# Shaming interrogatives: Admonishments, the social psychology of emotion, and discursive practices of behaviour modification in family mealtimes

**DOI:** 10.1111/bjso.12346

**Published:** 2019-11-12

**Authors:** Jonathan Potter, Alexa Hepburn

**Affiliations:** ^1^ School of Communication and Information Rutgers-The State University of New Jersey New Brunswick New Jersey USA

**Keywords:** admonishments, assessments, conversation analysis, child interaction, discursive psychology, descriptions, directives, emotion, epistemics, language socialization, morality, naturalistic data, parent, social development, shame, socialization

## Abstract

This paper contributes to the study of admonishments, the operation of shaming in family interaction, and more broadly presses the virtue of a discursive psychological reconsideration of the social psychology of emotion. It examines the methodological basis of contemporary research on shame in experimental and qualitative social psychology, illustrated through the Test of Self‐Conscious Affect (TOSCA) and qualitative work using shame narratives. Doubts are raised about how these methods can throw light on shaming practices in natural situations. The study uses a collection of video recordings of family mealtimes, focusing on admonishment sequences in which parents address the interrogatives ‘what are you doing’ or ‘what did I say’ to a ‘misbehaving’ child. Despite the interrogative syntax, rather than soliciting information we show that these interrogative forms pursue behaviour change by publicly highlighting both the problem behaviour and the child’s active and intentional production of that behaviour. This is the sense in which the practice can be understood as shaming. Although this practice prosecutes shaming, ways in which the children can ignore, push back, or rework parents’ actions are highlighted. This study contributes to a broader consideration of how enduring behavioural change can be approached as a parents’ project.

## Background

This paper introduces and contributes to a discursive psychological study of admonishments – rebukes, scolds, reproaches, and the like. These are a notable part of human life that have been little studied, despite their profound involvement in social influence practices and their displays of everyday morality in practice. At the same time, it contributes to discursive social psychological work on emotion, taking the example of family mealtime practices of ‘shaming’. The focus here is on how emotion is live in the conduct of participants, in avowals, attributions, displays, and orientations, rather than participants’ post hoc reports of conduct or speculations about how they would act in invented, prototypical, or otherwise idealized situations (e.g. Edwards, 1997; Edwards & Potter, 1992; Wiggins & Potter, 2017). Its theoretical basis lies in the work of Wittgenstein, Harvey Sacks, and Emanuel Schegloff, treating interaction as the primary site of human sociality, where actions unfold in an ordered sequential manner in ways that sustain their necessary intelligibility. We will consider how this plays out in contemporary social psychological research on emotion, and specifically shame. Our consideration of shame will move from ideas of shame as a state or experience to practices of shaming.

This work is part of a broader project on family mealtime conversations, childrearing interaction, and core practices through which one party, typically, but not always a parent, works to modify the behaviour of another, typically, but not always a child. This includes discursive and conversation analytic work on requests, directives, and threats (e.g., Craven & Potter, 2010; Hepburn & Potter, 2011a; Kent, 2012). More broadly, it contributes to an understanding of how enduring behaviour change, ‘socialization’, is being reconsidered from more interaction‐focused perspectives (Hepburn, forthcoming).

### Emotion and shame

Discursive social psychological (DSP) work on emotion has taken two broad forms. First, it has focused on the way emotion terms or tropes are flexibly and rhetorically used in avowals and attributions in naturalistic discourse, and the way they are fitted into broader discursive practices (Buttny, 1993; Edwards, 1997, 1999; Locke & Edwards, 2003). For example, in relationship counselling sessions descriptions can be built using notions of anger that display rationally grounded moral outrage, or rational grounding can be undercut in constructions that build anger as irrational and dispositional; alternatively the moral blaming work of avowing anger can be countered in constructions that replace ‘anger’ with ‘upset’ with the more psychological and individual framing that this brings (Locke & Edwards, 2003). The analytic point in DSP is not about the accuracy of such descriptions, or the psychological truth they may or may not capture; it is focused squarely on the practical role of emotion talk in human life. DSP maps the role of emotion language as it is rhetorically constitutive of unfolding actions; it figures in accusations and accounts, assessments, and compliments. It is live in everyday interaction among family and friends, and in institutional settings such as courtrooms, mediation sessions, and therapy.

A second focus of DSP is on emotion displays and orientations. For example, in her research on crying, Hepburn (2004) shows how the vernacular category ‘crying’ encompasses behaviour with a range of different features (including delay, lowered volume, elevated pitch, tremulous delivery, sniffs, sobs, etc.). These are features that sometimes inflect delivery and sometimes delay or terminate it. Moreover, speakers show a close orientation to these features, modulating their own pace, and volume of delivery in ways that both match the emotional stance and show attentiveness (Hepburn & Potter, 2012). On the NSPCC child protection helpline, for example, callers will apologize for disrupting reports of abuse, while call takers licence those disruptions (e.g. ‘take your time’). The category ‘crying’ is start point for research, but analysis reveals how this collection of phenomena is better seen as a loose but organized collection of elements and practices. It is notable that in most of the examples studied, there is no use of the word ‘crying’ itself; although like other terms in the emotional thesaurus it can be used in performative ways. The analytic challenge is to move beyond the category to reveal the organization of practices.

The research reported below takes this second approach; although it starts with the category ‘shame’, as with ‘crying’ it is using the category as a steppingstone to reveal a set of practices. The term ‘shame’ does not appear in these data. At the same time, ‘shame’ has been studied in very different traditions of social psychology and exploring the methodological procedures at the heart of these traditions of work on shame allow us to highlight important features of the DSP approach taken here.

### Methods for studying ‘shame’ and social psychology

We will show how shame is constructed as an analytic topic using one example from experimental social psychology and one from qualitative social psychology. The point is to highlight the methodic practices at the core of these studies and the way particular notions of shame are methodologically baked into the studies.

One of the most common instruments used by experimental social psychologists working on shame is the Test of Self‐Conscious Affect (TOSCA; e.g. Gilbert, 2000; Gousel & Leach, 2011). The instructions state:

Below are situations that people are likely to encounter in day‐to‐day life, followed by several common reactions to those situations. As you read each scenario, try to imagine yourself in that situation.

This is item one:

1. You make plans to meet a friend for lunch. At five o’clock, you realize you have stood your friend up.

**Table:** 

	not likely	very likely
a) You would think: “I’m inconsiderate.”	1‐‐‐2‐‐‐3‐‐‐4‐‐‐5	
b) You’d think you should make it up to your friend as soon as possible.	1‐‐‐2‐‐‐3‐‐‐4‐‐‐5	
c) You would think: “My boss distracted me just before lunch.”	1‐‐‐2‐‐‐3‐‐‐4‐‐‐5	

Aggregate values across the items give scores on ‘shame self‐talk’ (a), ‘guilt self‐talk’ (b), and ‘blaming others’ (c). Let us start with a series of observations about this test.

First, and fundamentally, note that although the aim is to generate measures of affect, the procedure is linguistic. The apparatus works through words (make plans, think, inconsiderate, distracted) and associated ‘paper’ scores.

Second, note that participants are not faced with a specific, actual event, but a prototypical, or scripted event. When filling in the score, the participant is speculating or theorizing about an emotional response of some kind that they *would* have in a situation characterized in a brief vignette. On the modal use of ‘would’ in specific practices, and its implications see Edwards (2006).

Third, there is a blurring of language and thought in the way the scores are described. Thinking is characterized by words and phrases. Thus you would think, in words, ‘I’m inconsiderate’ which is described in the scale as ‘self‐talk’. This is a Fodoresque vision of thought being literally formed in language (in this case English). Again, this is baked into the instrument – participants are not provided any alternative ways of talking about their thinking.

Fourth, although all of this is done through language (and numbers), it is shorn of an explicit functional or interactional context. There is no interaction with another party (except in the sense that these test questions have been invented and delivered in paper form by the researcher). There is no scope for repair, uptake, pushback, displays of understanding; nor is there timing, prosody, overlap, and the kinds of paralinguistic features that might be taken, in natural settings, to indicate emotional investment or disturbance.

Fifth, although there is no interactional context, the different responses could all be elements of discursive practices. ‘I’m inconsiderate’ might be part of an apology, which might be required in a situation where shame is relevant (see, e.g., Drew *et al.*, 2017). ‘I will make it up to you’ might indicate compensation is impending. And ‘my boss distracted me just before lunch’ might be part of a standard excuse or account. That is, these invented elements of discourse could occur in people’s practices; we simply do not know if there are these connections and how this is consequential when filling in the test. The point here is that the combination of instrument and analysis *reproduces rather than discovers* a cognitivist model of shame.

None of this is to suggest that the TOSCA is not interesting or that it does not capture different patterns of responding from different individuals. What we are emphasizing is that it is an instrument that sits in highly complex and so far, unexplicated relationship to participants’ actual practices. It purports to measure ‘self‐conscious affect’ but that is cashed out in terms of numerical values on generalized statements such as ‘shame self‐talk’. It does not explicate what shame is as an entity or the kinds of practices through which shaming is achieved or shame is oriented to.

Social psychologists from a very different tradition have adopted what may be glossed as a qualitative approach to shame. Again, we will work with an illustrative example to highlight methodological issues. Leeming and Boyle (2013) focused on the ‘management and repair of shame’ by recruiting participants from a university community and asking them to write an account:

…about experiences of shame which have taken place in front of one or more other people. Please describe the most recent situation you can clearly recall where you felt particularly shamed in front of other people, whether or not you think it was sensible to feel like this.

When telling the story of what happened, please provide as much detail as you can about the specific moment (if there was one) when you felt most shamed and the events before, during, and after this. (Leeming & Boyle, 2013, p. 144).

We will again offer a series of observations not designed to undercut Leeming and Boyle’s study but to crystalize some of its assumptions and show the value of a contrasting research approach.

First, unlike the TOSCA, their method allows participants to consider a specific event that actually happened to them, rather than a prototypical invented situation. They are recruited to talk about experiences of shame and they cooperatively build accounts with this frame. Note, however, that we do not know whether they would otherwise have used the category shame or whether they would have felt more comfortable or more accurate working with notions such as guilt or embarrassment. They can only work with the instructions offered.

Second, post hoc narrative reconstructions of this kind are subject to a range of difficulties. It is not just that people may misremember, invent, or reconstruct what went on. It is that they are describing an event from an individual perspective. The method centres on one individual’s partial version with no access to alternatives. The primacy of such a perspective helps bake cognitivism into the method.

Third, if we consider shame in situations of interaction then we are likely to be interested in interactional phenomena that are extremely hard to recall and represent accurately. People do not have good skills in capturing and representing features of prosody, overlap, stress, word order, and basic lexical choices. Even skilled copy typists working with concurrent recordings find this a challenge. These are all features that conversation analysis and discursive psychology highlight as fundamental to the specifics of interaction.

Fourth, although the participants were asked to provide as much detail as possible, all but one of the examples in the paper are glosses rather than even putative verbatim reports (even the exception has only two words of putative reported speech). Thus: ‘My mum later told me that my friend sounded very upset and angry that I’d let her down’ (Leeming & Boyle, 2013, 147). We are not told the mum’s words, although we are given categories upset and angry that she may or may not have used. Note here the authors’ ‘thematic analysis’ is working with a gloss (the undergraduate student’s gloss on an event) of her mother’s gloss on how a friend had sounded. There are a lot of descriptive, interpretive, and reconstructive layers of the world as it happens. Our point is not that the analysis offered is uninteresting; but it leads us to question its empirical traction for understanding practices.

Fifth, the analytic conclusions offered by the authors move between constructions of experience and observations about patterns of events. Many of these challenges are generic for social researchers working with elicited narratives of this kind. There are particular challenges in doing such work based on qualitative interviews and similar materials (Potter & Hepburn, 2005, 2012).

Our general point, again, with respect to both the TOSCA and the narrative‐based methods is not to dismiss these styles of work as inadequate, but to note that they leave an important and unpopulated space for research that starts with records of actual interaction, using conversation analytic tools that allow us to see psychological matters as parts of interactional practices. Discursive psychology has left the laboratory and the interview cubicle behind to instead look at such practices in the wild (Potter & Shaw, 2018; Stokoe, 2018). With respect to studying shame there is important work to be done on performative uses of the category in political disputes, say (“Boris Johnson – Man with no shame”). However, the approach taken here goes down a different route. It is focused on a type of admonishment which works through practices that can be understood as shaming while not using the word itself.

### Admonishments, interrogatives, and shaming

This study is part of a broader set of work on the role of admonishments in parents’ practices of modifying their children’s behaviour. This considers practices parents draw on to effect enduring behavioural change, including the use of moral categorizations (‘that’s naughty’) that label behaviour as not only inappropriate in the moment but also in future occasions, and targeted scolds (‘Anna!’) which can be precisely placed to mark conduct which (from the parents perspective) is in appropriate or impermissible.

In an earlier paper (Hepburn & Potter, 2011b), we suggested that these actions are organized into a cline, often moving from less controlling to more controlling actions. In more recent work, Hepburn (forthcoming) showed how this cline reveals an organizational preference for the recipient of behaviour management actions (the child) to self‐initiate the appropriate behaviour – that is, requests typically come before directives, directives before threats, and such actions are often interspersed by recycling turns, leaving silences, and cajoling moves. In this way, across sequences of talk, parents can be shown to be engaged in a socialization project. The aim of the current analysis was to elucidate one practice that arises when considering a particular method of behaviour management by parents, which we call a ‘shaming interrogative’. By elucidating the nature of the practice, we will be able to give a more precise interactional take on shame.

The current paper will thus concentrate on practices that use interrogative grammar to highlight what is (from that parents’ perspective) delinquent or unwanted behaviour. It will focus in particular on two forms – ‘what are you doing’ and ‘what did I say’ – and their variants. We are calling these interrogatives rather than questions because although they take the grammatical form of a question, the action they prosecute is not eliciting information. In our analysis we will consider what occasions such interrogatives, where they appear sequentially, how they work to publicly build the recipient as having been knowingly and actively delinquent (shaming), and some of the ways they are responded to.

## Method, materials, and participants

We draw on the discursive psychological approach to analysis (Hepburn & Potter, 2004; Hepburn & Wiggins, 2007), which utilizes the methods of conversation analysis (Sacks, 1992; Schegloff, 2007). From a conversation analytic (hereafter CA) perspective, our analysis contributes to work on actions performed by interrogative formats, notably polar questions (Raymond, 2003; Stivers & Hayashi, 2010), tag questions (Hepburn & Potter, 2010, 2011b), advice implicative interrogatives (Butler et al., 2010), and, perhaps most relevantly, account solicitations (Bolden & Robinson, 2011; Koshik, 2005). In her discussion of wh‐ questions used as challenges, Koshik (2005) notes that these types of question are not considered by recipients to be seeking information, but rather occur in the environment of ongoing disagreement or trouble, and convey a negative assertion, for example ‘how would I know’ conveys ‘I wouldn’t know’. We also contribute to a tradition of work on actions in family mealtimes (offers, requests, directives, and threats).

The primary analytic materials are a corpus of recordings of nearly 100 mealtimes recorded by seven families, each of whom was given a digital video camera and asked to record around 15 mealtimes. No researcher was present during recording, and the family was encouraged to only record when they were comfortable to do so. Effort was put into explaining the general aim and value of the research to all participants, especially the younger ones, and ethical permission to include anonymized extracts in research meetings and academic outputs was obtained from all participants. All families are middle class (both parents have professional occupations) and are either White or Asian British.

Reactivity has rarely been found to be a problem using naturalistic materials of this kind, where the focus is on commonplace but little researched conversational practices. For further debate on our use of qualitative materials, see Griffin (2007) and response by Potter and Hepburn (2007). A crucial issue for us is that shaming interrogatives emerge in real‐time and are unlikely to be recalled post hoc in self‐report style questionnaires of the kind used by Leeming and Boyle (2013).

A transcription service was used to generate a first‐pass orthographic transcript of each recording. A broad search of materials was used to build an initial corpus of candidate interrogatives, transcribed using Jeffersonian conventions (Hepburn & Bolden, 2017). Our focus at this time was not to assess prevalence but to gather a spread of examples to enable a description of the practice. Our experience as parents and through presenting the material in talks is that the practice is quickly recognizable in British, American, and Northern European households. We are trying to give a more systematic, analytically based account of a commonplace parenting practice. Although we present transcript, the analysis works with the video at all times and, where interactionally relevant, incorporates the role of gaze, gestures, and other embodied features (for a relevant overview of issues of using video in this way, see Heath, Hindmarsh, and Luff (2010).

### Analysis

In this section, we start to explore some features of the practice of issuing an interrogative in the environment of managing a child’s ‘problem’ behaviour. We will focus in particular on the roles of epistemics (how displays of knowledge are built and oriented to interactionally; Heritage, 2012; Heritage & Raymond, 2005) and intentionality. We then consider the range of ways these interrogatives can be responded to.

Before we begin, we will start by unpacking what admonishments look like in our corpus.

#### The practice of admonishing

Hepburn and Potter (2011b) traced out a distinction between threatening and admonishing using the following example, in which Mum has just fed Anna (3 years old) a forkful of food (Anna normally feeds herself). Mum threatens withdrawal of ‘pudding’ (lines 5‐6) if Anna doesn’t eat some of her dinner. In direct defiance of Mum’s threat, Anna spits out the food that she had in her mouth (line 8) and this is followed by what we see as classic admonishing actions built up from lines 9 onwards:

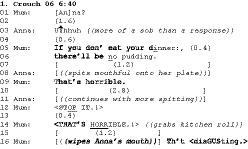

This analysis started to overview some of the characteristics of these admonishing actions, for example:

1. They are often focused on halting ongoing problem behaviour, although this focus may be combined with attempts to elicit conformity and/or contrition.
2. They negatively assess the child’s conduct, often with extreme or noticeably intensified formulations (Pomerantz 1986; Edwards, 2000) such as Mum’s ‘horrible’ and ‘disgusting’ here.
3. They contain prohibitions such as ‘no’ or ‘stop it’ (line 12).
4. Their sequential location is important, as is the prosodic delivery; for example, the repetition on line 14 targets not just the first iteration of problem behaviour on line 9, but also Anna’s defiance on line 11. The prosodic delivery with elevated volume, stretch, and emphasis, relative to line 9, allows the reiteration of the same moral categorization.
5. Their design and delivery can treat the child as intentionally misbehaving, continuing a course of action that has been categorized negatively and publically by the parent.

The characteristics that allow us to identify admonishments – sequential position, prosody, non‐vocal behaviours, assessments fitted to conduct – are very different to what is captured in the social psychological methods we reviewed above. Both structured TOSCA items and post hoc open‐ended reconstructions almost entirely fail to capture them. Note also that these admonishment practices are distinct from conversational repair – they are not designed to manage ‘problems of speaking, hearing and understanding’ (Schegloff, *et al.*, 1977, pp. 361) rather they are focused on modifying the recipient’s conduct. Having highlighted some common general features of admonishments, we will now focus on admonishing actions that use interrogatives of either the ‘what are you doing’ form or the ‘what did I say’ form.

#### Shaming interrogative format 1 – ‘what are you doing’

**Figure 1:**
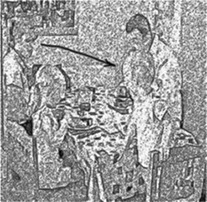
Anna and Mum.

In the following example from the family in the previous extract, Anna, who has so far not been eating, has been directed to eat by Mum (line 1) and responds with an upset/protesting noise on line 2. Also present at this mealtime is Katherine (5) and Dad – both are eating and attending to their food. Anna is kneeling sideways on her chair, looking at Mum throughout this extract; she holds herself steady with a hand on the table and chair and makes no attempt at further eating, although she does have food in her mouth. See Figure 1.

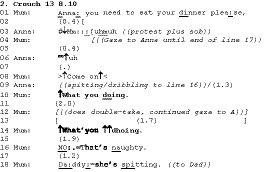

Following mum’s cajoling (08), Anna starts to dribble or spit, with small amounts of saliva coming out of her mouth between 4 and 17. Mum’s description of it as spitting on line 18 formulates Anna’s actions as deliberate. Mum’s interrogative ‘what are you doing’ is issued in an environment where Anna’s ‘spitting’ is public and clearly visible to her: Mum and Anna are both locked in mutual gaze from line 4 onwards. Mum’s posture and prosody (12‐16) display surprise, shock, and mounting anger.

This is not, then, an interrogative that projects knowledge asymmetry, with Mum needing Anna to tell her what she is doing: She has a clear view, she is looking intently at Anna, and Anna looking back can see that. Indeed, Mum is able to offer a category – ‘spitting’ – that would itself be the answer if her action was genuinely seeking information. Rather, this interrogative format treats the child as in a position to respond, therefore as knowingly doing something wrong (not eating, spitting, defying a directive). Through its interrogative form, it treats the recipient as able to judge that her conduct is not simply accountable but also problematic (‘naughty’, line 16). The interrogative form makes this public, putting a response requirement on the child. If Anna were to answer ‘spitting’ she would be, depending on delivery, self‐incriminating, in the sense that she would build herself as indeed deliberately spitting, or insolent, that is, as defiantly spitting. Mum’s construction here builds the child as both deliberately misbehaving and knowing that what they are doing is wrong. The public building of the recipient’s deliberation and knowledge is why we are calling these shaming interrogatives.

The repetition of ‘what are you doing’ (line 14) is similar to Extract 1 in its use of elevated volume, allowing a more emphatic reissuing of the same turn to address Anna’s non‐compliance: She continues to dribble/spit. Also, both extracts (lines 10, 13, and 15 in Extract 1, and lines 11 and 15 in Extract 2) have long transition spaces (Sacks, Schegloff, & Jefferson, 1974). As Hepburn (forthcoming) shows in greater detail, this cline in the building of admonishments offers Anna an opportunity to remedy her problem behaviour and at the same time sustains both hers and Mum’s focus on that problem behaviour.

We can contrast this exposure of problem behaviour in the transition space with the following extract, in which Lisa (9) and Ellie (6) are eating an evening meal with Mum. We join the extract as Mum is grating cheese onto Lisa’s plate (line 1). At the same time, Ellie is eating her peas with a tea strainer (line 2).

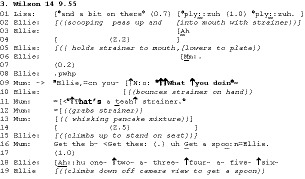

**Figure 2:**
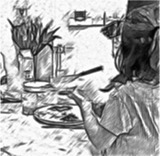
Mum takes strainer from Ellie.

Ellie’s problem behaviour is using a tea strainer to eat her peas. There are some differences here that we can contrast with Extract 2. First, note the lack of extended transition space following Mum’s issuing of ‘what you doin’ on line 9 (indeed it is latched to Mum’s subsequent verbal and embodied conduct on lines 11 and 12). We can contrast this with the extended silences on lines 11 and 15 of Extract 2. Second, note Mum’s reduced volume in delivering our target turn on line 9 – it comes off as if talking to herself as much as to Ellie, which contrasts starkly with the prosody in Extract 2 line 14. These two elements – the latching of more talk post‐interrogative, and the reduced volume, combine to reduce the response relevance of Mum’s turn on line 9. That is, there is less pressure on Ellie to inspect her own conduct, due to the lack of opportunity to immediately respond to the interrogative. Moreover, on line 11 Mum simply removes the source of the offending behaviour – the tea strainer – such that no continuation from Ellie is possible (see Figure 2). A further remedy to the problematic pea‐eating is provided by Mum on line 16 in the form of a directive to ‘get a spoon’, with which Ellie complies.

One way of understanding Extract 3 is that Mum indexes the relevance of a more shaming focused approach to Ellie’s behaviour in lines 9 and 11. However, perhaps because this is a fairly minor offence, Mum moves to physical intervention (removing the misused implement) and directs Ellie to get the correct implement. Ellie’s delayed compliance maybe marks her autonomy or partial resistance to Mum’s directive (cf. Hepburn & Potter, 2011b; Kent, 2012).

This parental move of not over‐exposing the source of trouble but instead simply removing it is also apparent in the following example in which Mum, Lanie (4) Finlay (15 months), and Dad are having dinner. Mum is telling Dad a story about a recent trip to the butcher’s. Lanie has pulled the butter dish over towards herself.

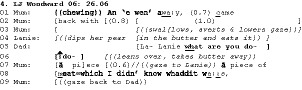

**Figure 3:**
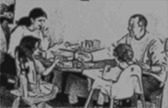
Dad takes butter.

On line 4, Lanie dips her pear into the butter, and in overlap with her placing the pear in her mouth, Dad issues a somewhat aborted version of our target turn on line 5. As we saw in Extract 3, his removal of the butter on line 6 effectively remedies the problem behaviour for Lanie – the opportunity for either compliance or non‐compliance is not given (see Figure 3). The whole episode occurs in parallel while Dad continues to be a recipient for Mum’s ongoing story. Indeed Dad’s interrogative is abandoned, possibly due to this overlap with Mum, but also perhaps due to the ease of remedying the trouble by simply removing the butter.

In our corpus, we find ‘what are you doing’ interrogatives as next actions to a child’s problem behaviour. We showed how the calibration of seriousness can be made through:

1. Underscoring the interrogative with elevated volume, stretch and/or pitch, or delivering it quietly;
2. Leaving a lengthy transition space versus continuing to speak post‐interrogative; and/or
3. Providing a material remedy by removing the source of the problem.

If the interrogative grammar was issuing a genuine question, it would mark an epistemic asymmetry between speaker and recipient, with the recipient able to provide the answer to the question being the required outcome. However, in each case here the parent’s interrogative targets the child’s visible conduct – spitting/dribbling, eating peas with a tea strainer, dipping food into the butter. The interrogative is not therefore targeting an answer that will increase the parent’s understanding. Moreover, none of our examples with this format get a verbal response; indeed, Anna’s response through lines 11‐16 in Extract 2 is embodied defiance – it continues or even exaggerates the delinquent behaviour. The role of this interrogative, then, is not to seek information but to manufacture a sequential position where the recipient is confronted with, and required to respond in some way to, their knowing and deliberate wrongdoing. We suggest that this public highlighting of deliberate wrongdoing, with its associated and challenging response requirement, has a role that we might gloss as shaming.

#### Shaming interrogative format 2: ‘what did I say’

While ‘what are you doing’ is more immediately responsive to the first noticing of problem behaviour, ‘what did I say’ follows one or more directive strategies that haven’t secured compliance. ‘What did I say’ is therefore more explicitly occupied with non‐cooperation, in addition to targeting the original problem behaviour.

The following example shows the importance of intentionality in problem behaviour – Anna’s (age 3) quiet, (seemingly) unintentional belching at the table (line 1) is subject to playful teasing by both parents – Dad on line 3 and Mum on line 12. In contrast, Katherine’s (age 5) loud intentional play belching on lines 19, 24, and 36 is treated as problematic. We can track the progression through different behaviour management strategies in 21 (prohibition), 26 (repetition with emphasis), and 38 and 41 (our target turns). These are followed by the threat (lines 45‐6).

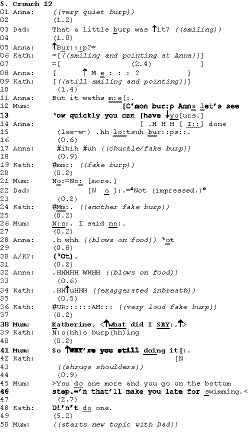

Here, Katherine not only ignores Mum’s attempts to halt her problem behaviour (fake belching) with repeated prohibitions in lines 21 and 26, as well as Dad’s similar turn on line 22, but she also produced a longer and much louder version of the same problem behaviour on 36. Her responses to Mum’s interrogatives show the problems and possibilities of responding literally to these as information seeking questions. The problem is that the response is self‐incriminating and doesn’t do appropriate contrition (indeed Katherine’s line 39 contains interpolated laughter, which recognizes that her utterance is in some way problematic and not to be taken literally, Potter & Hepburn, 2010). Katherine’s response then elicits a further account‐seeking interrogative from Mum ‘So why are you still doing it’ on line 41.

In their analysis of why‐interrogatives as ‘direct account solicitations’, Bolden and Robinson (2011) claim that where they occur in environments where the speaker has joint access to the event, they indicate a lack of ability to make sense of that event. Accounts from recipients provide a remedy or transformation of this state of affairs; hence, why‐interrogatives allow for the possibility of a satisfactory account for an event, while also communicating its inappropriateness. However, as we see here, responding to this question on 41 is again self‐incriminating for Katherine – there is no good account available, as Katherine’s shrug indicates. This then leads to the threat from Mum, a pattern analysed previously by Hepburn and Potter (2011b).

We can contrast the example above with the following, our only example of contrition in the form of apology. Mum, Charlie (5), and Jack (9) are having breakfast. Prior to this extract, Mum has told Jack to take his pills (he is diabetic) and Charlie has asked Mum to guess which tune he is humming. She is unsuccessful, but Jack guesses the tune and starts singing it on line 1:

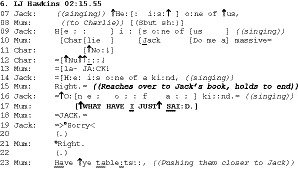

**Figure 4:**
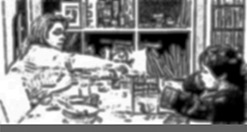
Mum reaching.

Jack hijacks Charlie’s humming game and then not only ignores Mum’s attempts to stop him at 10, 13, and 15 (see Figure 4 as Mum reaches over to Jack to secure his attention), but also continues singing over Mum’s shouted turn on 17 – our target interrogative. Following this he quickly apologises, although in a fairly minimal way (Heritage & Raymond, 2016). Singing at the table is not problem behaviour in itself, rather the problem seems to lie more in the interruption of Charlie’s game, and perhaps more importantly ignoring Mum’s attempts to restore order. Unlike our previous example, there is no attempt to reproduce what Mum actually said, rather an apology suffices to head off this sequence.

As we noted above, why‐interrogatives allow for the possibility of a satisfactory account for an event, while also communicating its inappropriateness. Here, ‘what have I just said’ seems to set up a similar requirement. The apology to Mum can be seen as an effective form of remedy for Jack’s transgression in contrast to Katherine’s literal answer in Extract 5.

## Discussion: Admonishments and interactional shaming

In this study, we have focused on a subset of admonishments in which parents use interrogatives – ‘what are you doing’, ‘what did I say’ and, ‘why are you doing it’ – in environments where (from their perspective) the child is behaving badly. Although issued with the syntax of a question, the practice does not pursue information; neither is an informational response typically forthcoming, without invoking further shaming, as we saw in Extract 5. The interrogative form nevertheless treats the recipient as relevantly able to answer the question. In the ‘what are you doing’ variant, the answer would specify their delinquent behaviour: spitting, eating peas with a tea strainer, dipping a pear into the butter. In the ‘what did I say’ variant, the answer would repeat the ignored prohibition: ‘no burping’, or directive: ‘take your pills’. In both cases, the interrogative draws attention to the delinquency, publicly treats the child as its conscious agent, and puts the child in a position of reporting on their own delinquency or defying the response requirements of the interrogative. Parents can exploit this by stretching the transition space (as in Extract 2 lines 11, 13, and 15) where the moral flaws have been highlighted but not yet responded to. It is the public, psychologically invasive, recipient challenging elements of the practice which lead us to use the term shaming.

Before moving to broader issues, let us consider shaming practices in terms of the immediate next action from the shamed individual. This focus is on the orientations they display and the behaviours they engage in. We will take them in an order that makes the pattern of possibilities clearer.

**Extract 6**: this is closest to what might be considered the preferred outcome of the shaming. Jack ceases his problem behavior (singing over Charlie’s humming game) and displays contrition with an apology. It is perhaps notable that Jack is the oldest target child in this collection. The potential for intriguing developmental questions is thus raised, pending a larger scale study.

**Extract 5**: here Katherine has repeatedly responded to directives to stop fake burping by doing further, louder fake burps. As we noted in our work on threats, as parents intensify their attempts at behavior management this provides a platform for increased resistance and defiance. Mum’s ‘Katherine, what did I say’ provides the opportunity for Katherine, like Jack, to suspend the problem behavior and show some form of contrition. Instead, Katherine uses this slot to do the direct answer to the question, resulting in an ‘account solicitation’ (Bolden & Robinson, 2011) ‘So why are you still doing it’, for which, again, there is no response that isn’t incriminating. Mum then moves up the behavior management cline by shifting from shaming interrogative to threat.

**Extract 4**: here Dad breaks off the shaming interrogative route and opts instead for rearranging the material scene, leaving Lanie unable to continue dipping pear into butter. Interactional contingencies play out in real time, Dad may well see success as ultimately more easily achieved by physical means and that pursuing the shaming project may be inappropriate for a minor infraction; it may also interrupt the focused conversation underway with Lanie’s mum. In the bustle of family settings, admonishments are regularly issued in parallel to unfolding projects of different kinds.

**Extract 3**: has some similarities to extract 4. There are problems in the production of the shaming interrogative and mum resorts to managing the physical setting, removing the tea strainer that Ellie was using to eat peas. Mum then directs Ellie to get a spoon to eat peas, and she complies with this.

**Extract 2**: here Anna builds the most defiant response to Mum’s ‘what you doing.’ She continues to dribble/spit while she and Mum are locked in eye contact for nearly 4 seconds (a long time where interactional parties are sensitive to delays of 0.2 seconds or less). Moreover, in the midst of this delay she does an ostentatious double‐take which displays to Anna that she is not just looking but that looking is taking in something problematic (Kidwell, 2005). Moreover, the dribbling/spitting is already a defiant alternative to Mum’s request: ‘you need to eat your dinner’. Again, this sequence moves through the cline of parenting behavior management options, with the moral attribution ‘that’s naughty’ and the passing of the parenting baton to Dad to address the delinquent behavior.

In each case, we can see the way the shaming interrogative is fitted to the task of behaviour management. However, it is not always (immediately and clearly) successful; it may be abandoned for simpler means or subject to push back from the recipient.

Let us consider implications, and offer some qualifications. First, this study is designed to highlight the existence of a practice and indicate some of its workings. It is not designed to show the prevalence of this practice, nor to link it to different social categories of families, to different age groups of children and parents, to different cultures or social groups, or to different outcomes immediate or long term. Identifying a practice of this kind is a precondition for such work and for stimulating novel research questions. For example, if we can identify shaming practices in this empirically grounded way, we could use them to ask cross‐cultural questions about morality and socialization. Shaming practices could be the equivalent of a genetic marker for a social psychology of culture. They might be prevalent in some cultures and absent in others. The more procedural, sequential focus shown here might allow refinement or rethinking of debates about shame vs guilt for example (Lo & Fung, 2011). They also encourage us to ask different kinds of questions about parenting and about children’s displays of understanding. It is likely that this broader work would allow us to refine our understanding of the basic shaming practices – there is an element of bootstrapping in CA research of this kind, but we have made a start located in a small collection of examples.

Take Extract 5 above where Katherine answers the ‘what did I say’ interrogative with ‘no burping’. Although this might appear to be a child treating the interrogative as a question, careful listening, and transcription picks out the interpolated particles of aspiration (‘laugh particles’) in Katherine’s turn, it is: N:o(hh)o burp(hh)ing. Work on such particles in words shows that they can be used to modulate the nature of an action (Potter & Hepburn, 2010). Here, Katherine’s delivery marks her understanding that her mother is not trying to recall her own injunction but pursue a different action.

Or take extract 4 where Dad’s ‘Lanie **wh** **at are you do‐ ↑** **do‐**’ is partially aborted in favour of simple physical removal of the problem. We might ask the question how much the modification reflects Dad’s local understanding of what will be effective with Lanie, or cause least disruption to other simultaneous projects, and possibly what she will understand in a way that will have a socializing effect on her future conduct. Thus, ‘what are you doing’ might be an index of the parent’s attributional understanding of what their child is capable of. Parents are calibrating their behaviour in real time to the attention, competences, and contingencies of their children.

We noted at the start of this paper that when social psychologists consider emotion they overwhelmingly focus on ‘experience’ or ‘feeling’ and yet their methods mostly approach experience by way of some verbal or written instrument. In the case of shame, we have illustrated this with the TOSCA and the open‐ended narrative recall task – both start with linguistic categories and descriptions, and infer experience from them. DSP remains agnostic about the referential adequacy of the cognitive and emotional thesaurus (Edwards & Potter, 2005). The discursive psychological issue is not what is happening under the skull, in the autonomous nervous system or wherever – it is how these concerns are managed in the practices of participants. Thus, work on emotion becomes interested in how emotion terms can be used in avowals, attributions, complaints, and so on, and how emotion displays can be managed and responded to. This is not the only position that social psychologists can take, of course, as the history of the discipline shows; however, we strongly argue that it is one way of doing social psychology that puts the social at its core. It works from social practices embedded in the normative organizations of conversation and interaction.

With respect to shame, we have held off speculation about how Katherine, Anna, Ellie, Lanie, and Jack are feeling. Are they feeling shame? How can that question be answered? Our discursive psychological approach has been to bracket off that question in favour of considering practices that publicly highlight reprehensible conduct in such a way that the child is treated as knowingly engaging in it, while having a requirement to respond. This is what we are calling shaming. Considering practices allows us to focus on next actions and how they throw light on what is going on. It is worth highlighting some nuances here. Unlike other work on shame, we are not trying to reveal its underlying nature, its cultural specificity, its contrast to guilt, and so on. Rather, we are trying to unpack the specifics of two closely related practices used to do admonishment. Ironically, approaches using interview questions, ethnographic field notes, or fixed choice items may seem closer to the nature of shame than this more direct study, but only because each bakes the very category shame into their findings.

One of the interests motivating the study of admonishments is better to understand socialization as a practical task for parents. Part of what we see in the collection are more or less successful attempts at immediate behaviour management. The ‘what did I say variant’ combines a focus on the behaviour that was the target of the original complaint, and the failure to comply with the parent’s injunction. The ‘what are you doing’ and ‘why are you doing it’ variants of the shaming interrogatives treat the child as able to provide answers to a question about their incriminating behaviour, so as knowingly engaging in wrongdoing, and thereby able to self‐correct in both this (relevant to immediate behaviour management) and future (relevant to socialization) instances. There is much to be done on the systematic organization of admonishment practices and, for instance, what might occasion one form of admonishment rather than another.
